# Estradiol use in the luteal phase and its effects on pregnancy rates in IVF cycles with GnRH antagonist: a systematic review

**DOI:** 10.5935/1518-0557.20170046

**Published:** 2017

**Authors:** Lanna Marla Andrade Pinheiro, Priscilla da Silva Cândido, Tássia Camila Moreto, Wanessa Gonzaga Di Almeida, Eduardo Camelo de Castro

**Affiliations:** 1 Infertility Clinic, Faculty of Medicine, Catholic University of Goiás, Goiânia, GO, Brazil

**Keywords:** luteal phase, estradiol, *in vitro* fertilization.

## Abstract

For all the steps of *in vitro* fertilization to occur successfully, factors such as the quality of retrieved oocytes and endometrial receptivity to the embryo must be ensured. Current studies have shown that endometrial receptivity can be optimized using dedicated exogenous progesterone for luteal phase support in assisted reproduction cycles. But it has not yet been established the benefits of additional use of estradiol in this support. Analyzing pituitary suppression protocols that employ GnRH antagonists, this review will address literature publications between the years 2000-2016, shedding light on this issue to answer questions about the benefits of supplementation.

## INTRODUCTION

In assisted reproduction cycles, the use of short protocol with GnRH antagonist, when compared to the long protocol of ovarian stimulation with GnRH agonist (GnRHa), presents corpus luteum hormone profile changes. In antagonist cycles; serum progesterone levels in the luteal phase may be higher than normal, and there may be a decrease in serum estradiol. This enables to infer a possible benefit from the estradiol supplementation, which can be larger in antagonist cycles, when compared to GnRHa cycles (Tavaniotou *&* Devroey, 2006).

In addition to the hypothesis that argues in favor of pituitary suppression using the GnRH antagonist, recent studies address what factors act as determinants of better results in the supplementation of the luteal phase. In this sense, although the benefits of luteal phase supplementation with progesterone have already been proven, the data on the benefits of additional supplementation with estradiol still need better evaluation so that it can be safely applied (Lukaszuk *et al.*, 2005; Kwon *et al.*, 2013). 

It has been recently suggested that the benefits of this additional supplementation is most evident when applied after the use of ovarian inhibition protocols with the GnRH antagonist (Tavaniotou *&* Devroey, 2006). However, data on this association require further analysis in the literature.

To elucidate the benefits of this supplementation in the luteal phase, this study aims at performing a systematic review of the literature to assess the effects of the use of estradiol on pregnancy rates in *in vitro* fertilization cycles. 

## METHODS

We carried out a systematic review of the literature with the following descriptors: "luteal phase", "estradiol" and "*in vitro* fertilization". The databases for consultation were PubMed, Latin American and Caribbean Literature (Lilacs) and the Scientific Electronic Library Online (Scielo). The papers searched were published in Portuguese, English and Spanish, from January 2000 to December 2016. The studies were selected by two researchers independently and blindly. When there was disagreement, a third researcher was asked for his/her opinion.

We included randomized clinical trials which used the GnRH antagonist protocol, comparing the luteal phase support with progesterone alone and estradiol with progesterone. The women in the papers had to be younger than 39 years, have a BMI between 18 and 29kg/m^2^, have intact ovaries, and hormonal profile with estradiol ≤80pg/mL and FSH ≤10.

We excluded the studies using long GnRH agonist protocols, those including patients with male factor infertility, poor responders to hormones or those suffering from polycystic ovary syndrome.

**Figure 1.**
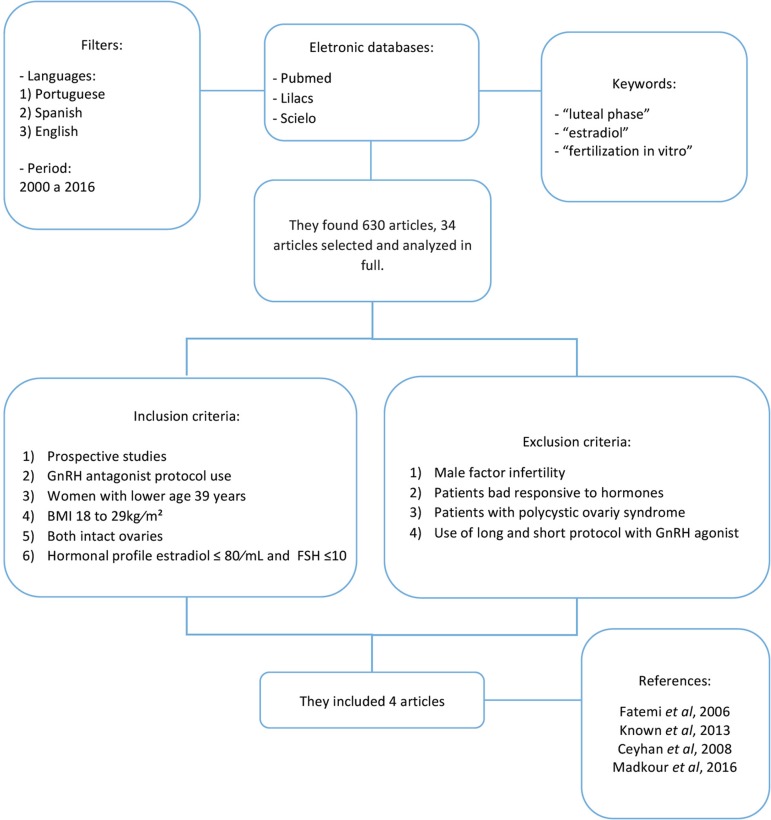
Methodology used in the systematic review construction.

During the search, we found 630 papers published, considering the descriptors and filters used in combination. After reading the abstracts, 34 papers were selected for full reading, of which only four matched the inclusion and exclusion criteria. The study analysis is depicted in the diagram below (Figure 1).

Since this is a review of scientific papers, it was not necessary to have the Research Ethics Committee's approval.

## RESULTS

The identification data is summarized in Table 1 and, the main data extracted from the results in each selected systematic review is summarized in Table 2, for comparative analysis. The main characteristics of the populations in each study are described in Table 3.

**Table 1:** Systematic review of the identification data.

Reference	Year	Local	Population	Dose (mg/day)	
				P	P/E
Fatemi *et al*.	2006	Spanish	201	600	600/4
Ceyhan *et al*.	2008	Istanbul	60	600	600/100*
Kwon *et al*.	2013	Seoul	110	90	90/4
Ismail Madkour *et al*.	2016	Multicentric	220	90	90/4

* µg/day, twice a week

**Table 2:** Presentation of the results found in the systematic review.

Reference	Pregnancy rate (%)			Implantation rate (%)		
	P	P+E	P-value	P	P+E	P-value
Fatemi *et al*. 2006	32.6*	28.9*	0.633	37.8	42.4	0.548
Ceyhan *et al.* 2008	61.9	56.5	0.72	†	†	†
Kwon *et al*. 2013	37.0	48.1	‡	15.8	26.0	0.035
Ismail Madkour *et al*. 2016	39.09	43.63	0.3	19.25	23.44	0.2

* Ongoing pregnancy per embryo transfer;

† Data not provided by the study;

‡ Not Significant.

**Table 3:** Patient characteristics in the studied groups.

Characteristic		Fatemi *et al*. 2006			Ceyhan *et al*. 2008			Kwon *et al*. 2013			Ismail Madkour *et al*. 2016		
		P	P+E	*p-value*	P	P+E	*p-value*	P	P+E	*p-value*	P	P+E	*p-value*
No. of patients		100	101	-	29	30	-	55	55	-	110	110	-
Age of patients (y)		32.05±3.66	32.03±3.55	NS	30.9±3.5	31.4±2.6	0.14	37.3±3.6	36.7±3.5	NS	30.23±4.13	31.11±3.23	0.07
Infertility duration (m)		-	-	-	-	-	-	48.5±24.5	45.3±26.8	NS	5.51±0.60	5.39±0.61	0.1
Body mass index (kg/m2)		22.7±2.76	22.0±2.82	NS	22.5±1.3	22.9±21.3	0.16	21.8±1.9	21.6±2.0	NS	22.51±6.42	21.97±5.74	0.5
No. of oocytes		12.3±7.40	11.9±6.15	NS	10.2±3.5	12.0±3.6	0.20	12.8±2.6	13.1±3.0	NS	11.6±2.34	12.2±2.28	0.6
Basal serum FSH (mIU/L)		<10	<10	-	5.6±1.1	5.2±1.1	0.14	7.2±1.9	7.0±2.0	NS	6.41±2.42	6.25±2.98	0.7
EI*%	Male factor	61	62.4	NS	6.9	13.3	-	36.4	34.5	NS	58.2	56.4	0.9
	Tubal/ peritoneal	22	19.8	NS	13.8	30.0	-	40.0	38.2	NS	24.5	21.8	0.6
	Others	4	4	NS	-	-	-	10.9	12.7	NS	-	-	-
	Unexplained	13	13.9	NS	79.3	56.7	NS	12.7	14.5	NS	-	-	

Values are presented as mean ± SD or number (%); E2, estradiol; P, progesterone; FSH, follicle-stimulating hormone; NS, not significant;

* Etiology of infertility; - Values awaiting in their articles

Of the four selected, the oldest study involved 201 patients and was published in the year 2006. The most recent one had a total of 220 participants submitted to intracytoplasmic injection cycles of sperm (ICSI) and was published in the year 2016.

One paper was excluded from this systematic review because it did not define which patients used the different types of ovarian stimulation protocol. 

## DISCUSSION

The ideal hormone combination for luteal phase support, the dose of medication and the right time of the cycle for the use of hormones is controversial information in the literature (Aboulghar, 2009).

About that a meta-analysis published in 2015 demonstrates that addition of oral estradiol during the luteal phase does not improve IVF/ICSI outcomes, even with different daily doses or with different routes of administration: oral, vaginal, and transdermal. Fifteen relevant randomized controlled trials were identified (included a total of 2406 patients), but concluder there was no statistical difference when estradiol and progesterone were used in the luteal phase support (Huang *et al.*, 2015).

Contradicting this, a meta-analysis published in 2015, analysed on the efficacy of progesterone versus progesterone plus estrogen of any form for luteal phase support during IVF. A total of 11 articles were included. Results of statistical analysis indicated that progesterone plus estrogen treatment was more likely to result in clinical pregnancy than progesterone alone (pooled odds ratio 1.617, 95% confidence interval 1.059-2.471; *p*=0.026). No significant difference between the 2 treatment regimens was found for the other outcome measures. As the authors showed, a risk of bias was present given that none of the articles addressed or performed blinding. Potential limitations of this study include the limited sample size (1756 subjects), the inclusion of different forms and dosages of estrogen supplementation, and the inclusion of subjects who contributed more than 1 cycle to a study (Zhang *et al.*, 2015).

Fatemi *et al*. (2006), in a prospective, randomized study of 201 women with normal response to gonadotropins evaluated in IVF cycles GnRH antagonist additional supplementation of estradiol to progesterone in the luteal phase. Two groups were defined, one with 100 patients, 90 of them were subjected to embryo transfer and received 600mg of progesterone vaginally. Another group of 101 women, 92 were undergoing embryo transfer receiving 600mg of progesterone associated with 4mg estradiol valerate per day. Without significant differences between groups of patients, it was found that implantation rate per embryo transfer was 37.8% for the group receiving only progesterone *vs.* 42.4% for the group that received progesterone and estradiol (*p*=0.548, not significant). Regarding pregnancy per embryo transferred the rate was 28.9% in the group that used only progesterone versus 32.6% in the group using progesterone and estradiol (*p*=0.633).

Like this, the authors concluded that the probability of pregnancy is not increased when it was added 4mg of estradiol to progesterone in the luteal phase support. To minimize the possible bias in this study, a fixed dose of recombinant FSh (rFSH) and a fixed GnRH antagonist protocol was used. Moreover, all embryos were transferred on day 3, and randomization performed by the number of embryos transferred. The choice of supplementation with 4mg of estradiol in the present study was randomized.

Ceyhan *et al*. (2008), in another prospective, randomized study of 60 women with normal response to gonadotropins and primary infertility, demonstrated that in IVF cycles with rFSH and fixed multidose GnRH antagonist, the additional estradiol supplementation with progesterone, supplemented group compared to only progesterone was not significantly increased (*p*=0.72) in pregnancy rates (56.5% *vs.* 61.9%).

In this study, the luteal phase support was performed from the first day after the capture of the oocyte until the eighth week of pregnancy and are used 600mg/day of micronized progesterone in both groups, and 100mg/day, 2x/week, transdermal estradiol in the test group. In addition to the small sample size, recognized by the authors, one of the study bias was that pregnancy rates presented in the study are overestimated because patients with poor quality embryos were canceled prior to embryo transfer, since the public assistance infertility in Turkey is limited to only three cycles per couple.

Kwon *et al*. (2013) presented a randomized prospective study, where the luteal phase support was started after the capture of oocytes. This study included 110 women of a university clinic of infertility Seoul. It was demonstrated that in cycles with GnRH antagonist adding estradiol to progesterone for luteal supplementation compared to isolated use of progesterone increased significantly (2.0% *vs.* 15.8%, *p*=0.035) embryo implantation rate in infertile patients who underwent IVF/ICSI. Furthermore, this supplemental use significantly (7.4% *vs*. 27.8%, *p*=0.010) reduced the incidence of vaginal bleeding luteal.

In this study, the luteal phase support was made from the capture of oocytes being used to support, in both groups 90mg/day of vaginal progesterone (Crinone 8%) and the test group received in addition 4mg/day of estradiol valerate orally until confirmation of pregnancy. Already progesterone was used until the tenth week of pregnancy. Despite the superior results of the test group compared to the rate and implementation, in the category pregnancy rate per cycle, there was no statistically significant increase (48.5% *vs*. 37.0%, *p*>0.05).

Ismail Madkour *et al*. (2016) in a more recent study, also prospective, randomized, 259 patients agreed that there are no benefits in relation to the luteal phase supplementation with additional use of estradiol with progesterone in ICSI cycles. For within these 259 patients, 220 were suitable for inclusion criteria, using the GnRH antagonist protocol for ovarian stimulation. It is noteworthy that, there was no significant difference between patients who were divided into two number of groups equal to 110, while group 1 received vaginal progesterone 90mg per day and the second group received, in addition to progesterone, 2mg of estradiol twice per day. As a result it was found that pregnancy rates per embryo transfer showed no significant difference between Group 1 (39.09%) and 2 (43.63%) (*p*=0.3). Likewise, ongoing pregnancy rates per embryo transfer conferred no significant difference with group 1 with 32.7% and 32.7% in Group 2 (*p*=0.1). Another fact that article provides us is that there was no significant difference in implantation rates and abortion rates.

The authors note that the results found in the article is related to protocols with GnRH antagonist to exist, then the need for evaluation cycles protocols with long GnRH agonist. Besides that, there is to search through large-scale trials and analysis target, the role of estradiol in the luteal phase supplementation in IVF/ICSI and dose for the same.

One of the biases to be addressed for successful luteal phase support is the ideal day cycle beginning. Studies show that very early high progesterone levels in the luteal phase, tend to lower pregnancy rates. Therefore, executing a late luteal phase support, one obtains improved synchronization between embryo and endometrium while embryo transfer is performed. Minor pregnancy rate is attained earlier in patients who received progesterone dose (day 2 or 3) in patients who received during late stage (Day 4 or 5).

## CONCLUSION

Only one study suggests the most successful embryo implantation in patients undergoing additional supplementation of estradiol with progesterone for luteal phase support in IVF/ICSI cycles used ovarian stimulation protocol with GnRH antagonist.

However, this success is not confirmed in any of the selected studies on pregnancy rate. Therefore, emphasizes the importance of further studies in order to clarify the role of estradiol in the luteal phase support in IVF cycles.
